# iFlow: A Graphical User Interface for Flow Cytometry Tools in Bioconductor

**DOI:** 10.1155/2009/103839

**Published:** 2009-11-12

**Authors:** Kyongryun Lee, Florian Hahne, Deepayan Sarkar, Robert Gentleman

**Affiliations:** Program in Computational Biology, Division of Public Health Sciences, Fred Hutchinson Cancer Research Center, 1100 Fairview Avenue N M2-B876, P.O. Box 19024, Seattle, WA 98109-1024, USA

## Abstract

Flow cytometry (FCM) has become an important analysis technology in health care and medical research, but the large volume of data produced by modern high-throughput experiments has presented significant new challenges for computational analysis tools. The development of an FCM software suite in Bioconductor represents one approach to overcome these challenges. In the spirit of the R programming language (Tree Star Inc., “FlowJo,” http://www.owjo.com), these tools are predominantly console-driven, allowing for programmatic access and rapid development of novel algorithms. Using this software requires a solid understanding of programming concepts and of the R language. However, some of these tools|in particular the statistical graphics and novel analytical methods|are also useful for nonprogrammers. To this end, we have developed an open source, extensible graphical user interface (GUI) *iFlow*, which sits on top of the Bioconductor backbone, enabling basic analyses by means of convenient graphical menus and wizards. We envision *iFlow* to be easily extensible in order to quickly integrate novel methodological developments.

## 1. Introduction

The analysis of large and highly complex datasets produced by modern high-throughput biomedical research can be a daunting task. Programmatic approaches, batch processing, and targeted analysis pipelines are typically employed to deal with this growing complexity. These solutions usually require considerable programming proficiency, or a rigid workflow structure that can be bundled into a static pipeline.

Flow cytometry (FCM) is an important emerging technology in immunology, cancer research, and health care. The technology is extremely versatile, and a multitude of different applications have been developed, which is reflected in a complicated and multilayered data analysis process. The analysis of FCM data has traditionally relied heavily on manual decision-making, and FCM software platforms typically present an interactive graphical user interface (GUI) as their primary interface [1, 2].

However, the sheer volume of data in high-throughput FCM experiments makes it impossible for an expert to efficiently perform fully manual analyses, and a certain degree of automation has become essential [3–5]. In the Bioconductor project [6], we have implemented a set of flexible command-line tools to facilitate the analysis of complex FCM data [7, 8]. The goal of the software is to foster the development of novel analytic methods by providing an open and extensible research platform that enables collaboration between bioinformaticians, computer scientists, statisticians, biologists, and clinicians. In order to succeed in this goal, we need to engage experienced practitioners who do not necessarily have programming skills. *iFlow* is a cross-platform software application meant to expose the tools and methods available in the Bioconductor project to such an audience by means of an interactive, extensible, and locally customizable GUI.

## 2. Results

**Figure 1:**
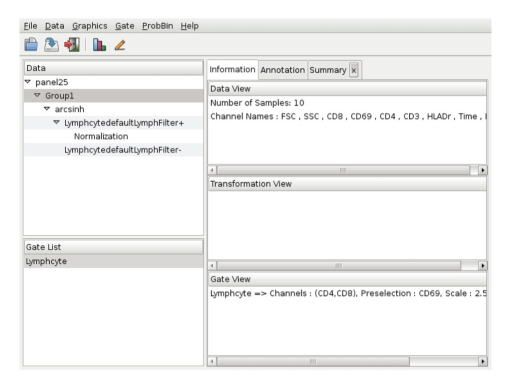
iFlow's main application window. Details on the components are given in the main text.

**Figure 2:**
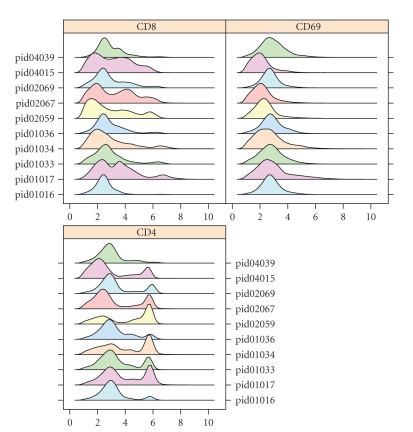
An iFlow graphics window displaying data of three FCM channels in the form of stacked density plots for ten different samples.

*iFlow* is implemented using the Gtk2 toolkit [9, 10], and sits on top of R and Bioconductor. It allows convenient management, visualization, and analysis of FCM data. On startup, *iFlow* will open an application window (Figure 1). Subsequently, one or more additional graphics windows may also be opened (Figure 2). The application window consists of a control panel and the main panel. The control panel lists all available datasets and gates, and allows the user to select one. All operations are peformed on the currently selected dataset. The main panel consists of a notebook with three types of tabs: *Information*, *Annotation*, and *Summary*; their contents are context-dependent.

The *Information* tab provides details about the currently selected data-set. It also displays information about previously defined gates and transformations, which can be reused in other tasks. The *Annotation* tab provides phenotypic information about the individual samples in the current data-set. It is possible to subset based on these covariates. Various summaries of the data can be displayed in additional *Summary* tabs.

A range of visualization methods are available from the *Graphics* menu. These include contour plots, density plots, scatter plots, ECDF plots, histograms, parallel coordinate plots, *Q*-*Q* plots, scatter plot matrices, and time series plots. Typical gating operations like rectangular and polygonal selections are available from the *Gate* menu. *iFlow* also supports basic interactive drawing of gates. In addition, a number of automated gating algorithms are available, offering data-driven selection of distinct cell populations. More general data manipulation operations are available in the *Data* menu, including various transformation options and data subsetting based on previously defined gates.

### 2.1. A Sample Session

Detailed usage instructions for *iFlow* are available in the manual accompanying the package. Here, we highlight some of the features one might use in a typical session. Data in the form of FCS files or R binary data files can be read in using the File|Load menu item. This step adds one or more data entries to the *Data* tab in the control panel. Selecting one such entry brings up a brief description of the associated dataset in the *Information* tab, as well as a tabular view of the sample covariates (e.g., Group ID, Patient ID, Visit number, etc.) in the *Annotation* tab.

As a next step, we may wish to create a new data-set with the subset of samples from a particular patient group. To do this, we first select the appropriate rows in the *Annotation*, and use the Subset item in the context menu that can be brought up using the right mouse button. Alternatively, one can use the Data|Subset By|Sample Covariates menu item. This creates a new data entry in the control panel that is nested within the original dataset.

We can next inspect the data graphically using items in the Graphics menu. A useful overview is given by stacked density plots (Figure 2). Such inspection may indicate the need to transform the data, which can be achieved using the Data|Transformation menu item.

The usual next step is to select a specific cell subtype for further analysis, for instance lymphocytes. Various types of gates can be created using the Gate|Create menu item; the list includes a *Lymphocyte gate* which tries to automatically select lymphocytes given a pair of channels and a third preselection channel. Once a gate is created, it can be applied to any dataset, and the results summarized in a *Summary* tab.

In large experiments, there is often a need for normalization before comparisons can be made across samples. The Data|Normalization menu item provides access to several normalization methods. As when creating subsets or transformations, normalization leads to the creation of a new dataset nested within its parent.

A video of *iFlow* demonstrating the above steps is provided as Supplementary Material to this manuscript (see Supplementary Material available online at doi: 10.1155/2009/103839). 

## 3. Discussion

Most FCM software implemented in the Bioconductor project was developed to address the growing need for automation in the data analysis process. However, command-line driven tools exclude a large group of potential users who are more familiar with GUI software. Moreover, in the course of working with high-throughput FCM datasets, it has become apparent that complete automation is not yet a practical goal, and some degree of manual interaction is crucial. We developed *iFlow* in order to make our methods accessible to a broader audience, and to combine the advantages of automated or semiautomated analysis with interactive data analysis.

The *iFlow* package contains all the code necessary to create and run the GUI, but it does not contain any code for the analysis of FCS data. Rather, it relies on functionality implemented in other R packages, which are installed and loaded at the same time as *iFlow*. It currently provides access to data visualization, manual and automated gating, transformations and basic data manipulations. This is sufficient for initial exploratory data inspection, as well as for prototyping large analysis projects.

Some of the capabilities exposed by *iFlow*, such as automated gating, already go beyond what is available in standard FCM GUI software. However, the primary long-term advantage of our software is its open and extensible nature. Additional functionality may easily be added in response to user feedback, or once common use cases have emerged. FCM is a field of active research, and we expect many novel analytical methods to be developed in the future. It would be relatively simple to incorporate these new methods into *iFlow* once they are implemented within the R/Bioconductor framework. This involves modification of the appropriate menu items and association of a particular R command with the extended menu. In this way, the extensive facilites already provided by various R add-on packages can be leveraged to expand the capabilities of *iFlow* with little additional work; for example, FCM data stored in relational databases could easily be imported using existing R packages for database access.

We believe that *iFlow* can serve as a useful interface for advanced statistical processing of FCM data, and that it will help bridge the gap between bench scientists, statisticians, and FCM data analysts. It is available as an R package on Bioconductor (http://www.bioconductor.org).

## Supplementary Material

Supplementary Material contains a video for the iFlow.
